# Increasing Neural-Based Pedestrian Detectors’ Robustness to Adversarial Patch Attacks Using Anomaly Localization

**DOI:** 10.3390/jimaging11010026

**Published:** 2025-01-17

**Authors:** Olga Ilina, Maxim Tereshonok, Vadim Ziyadinov

**Affiliations:** Science and Research Department, Moscow Technical University of Communications and Informatics, 111024 Moscow, Russia; o.v.ilina@mtuci.ru (O.I.); v.v.ziyadinov@mtuci.ru (V.Z.)

**Keywords:** adversarial patch attack, robustness, pedestrian detection, deep convolutional neural network

## Abstract

Object detection in images is a fundamental component of many safety-critical systems, such as autonomous driving, video surveillance systems, and robotics. Adversarial patch attacks, being easily implemented in the real world, provide effective counteraction to object detection by state-of-the-art neural-based detectors. It poses a serious danger in various fields of activity. Existing defense methods against patch attacks are insufficiently effective, which underlines the need to develop new reliable solutions. In this manuscript, we propose a method which helps to increase the robustness of neural network systems to the input adversarial images. The proposed method consists of a Deep Convolutional Neural Network to reconstruct a benign image from the adversarial one; a Calculating Maximum Error block to highlight the mismatches between input and reconstructed images; a Localizing Anomalous Fragments block to extract the anomalous regions using the Isolation Forest algorithm from histograms of images’ fragments; and a Clustering and Processing block to group and evaluate the extracted anomalous regions. The proposed method, based on anomaly localization, demonstrates high resistance to adversarial patch attacks while maintaining the high quality of object detection. The experimental results show that the proposed method is effective in defending against adversarial patch attacks. Using the YOLOv3 algorithm with the proposed defensive method for pedestrian detection in the INRIAPerson dataset under the adversarial attacks, the mAP50 metric reaches 80.97% compared to 46.79% without a defensive method. The results of the research demonstrate that the proposed method is promising for improvement of object detection systems security.

## 1. Introduction

The development of automatic neural-based object detection in images has revolutionized the field of computer vision. Such systems are widely used in various fields of human activity, such as medicine [1], pedestrian detection [2,3], autonomous driving [4], video surveillance systems [5], analysis of urban changes [6], and search and rescue operations [7].

The ability of neural network systems to identify and localize objects in images opens new possibilities for automating tasks that previously required human participation. In this work, YOLOv3 is used as an object detector [8] because it is the most commonly used approach to solve the problem of detecting multiple objects in a single image with a sufficient detection rate. This architecture acquired the greatest popularity for detecting objects in a video stream because of its rate of operation. It is also worth noting that the source code and the weights of the model trained on the extensive MS COCO dataset [9] are publicly available. This factor increases the attractiveness of using this model to solve the problem of object detection and makes possible comparison of the obtained results with the global level.

**Figure 1:**
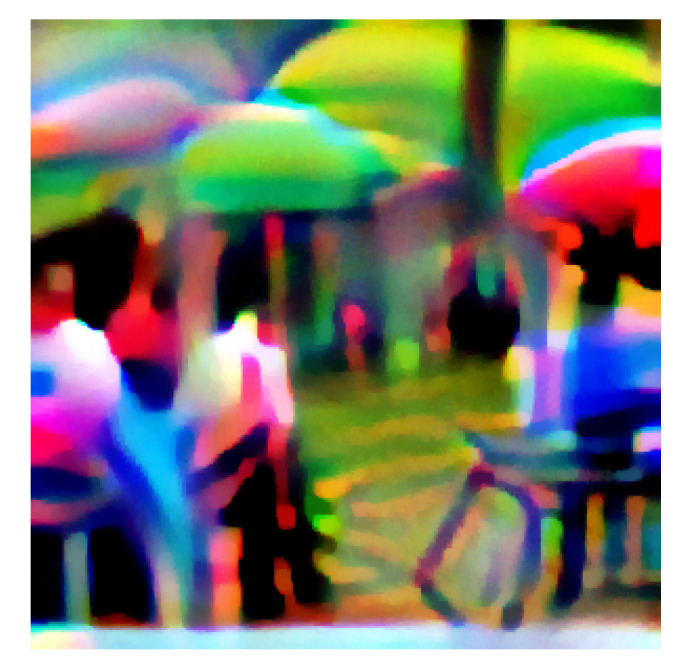
Example of an adversarial patch attack, generated by minimizing the object detector’s objectness score. The image is taken from [13].

However, despite significant progress in the development and application of such systems, they face a serious threat from adversarial attacks [10]. In this paper, white-box adversarial attacks are considered as they are more effective than analogues of black-box adversarial attacks [11]. Adversarial attacks can be divided into digital and physical implementations. Digital attacks directly manipulate the pixel values of the input images, which implies full access to the images. This type of attack can be implemented exclusively in digital form. Physical attacks introduce real-world objects into the environment that can affect the output of neural network systems. Real-world attacks, typically, are small fragments of images (patches), which are optimized in a digital environment before printing [12]. A prominent physical patch attack implementation is presented in the work [13]. The authors of [13] described an attack in a digital format that can be printed and applied in the real world. These patch attacks are aimed at deceiving the YOLOv3 system, pre-trained on the MS COCO dataset. The authors in [13] optimized adversarial patch attacks on the INRIA Person training dataset [14] to hide pedestrians. This dataset was created to solve pedestrian detection problems and contains images of people. The INRIA Person dataset contains 614 images for training and 288 images for testing. An example of the patch noted above is shown in Figure 1.

Such attacks aimed at fooling neural networks can lead to critical errors in various areas. For example, in autonomous driving, object recognition faults can have catastrophic consequences. Adversarial patch attacks have demonstrated their effectiveness in misleading traffic sign classification systems [15], and making pedestrians [13] or even cars [16] undetectable.

The main contribution of the paper is summarized below. The novel high-performance defensive method based on anomaly detection achieves significantly better resistance to adversarial patch attacks. The proposed method includes an unsupervised deep convolutional neural network and extensive yet computationally compact statistical analysis.

In this paper, we propose a new anomaly localization-based method to reduce the impact of adversarial patch attacks on the object detector. The remainder of this paper is structured as follows: Section 2 provides a brief overview of existing methods for removing adversarial patch attacks in images. Section 3 describes the proposed method. Section 4 demonstrates the implementation details and evaluation metrics of the state-of-the-art defense methods. Section 5 presents the results and discussions.

## 2. Related Work

One of the most popular methods of adversarial attack counteraction is adversarial training [17,18,19]. This method increases the model’s reliability by augmenting the training data with adversarial images. A different approach was proposed in [20]. The authors suggested adding a new class (namely, a class with an adversarial patch attack) and retraining the used object detector on a dataset enriched with adversarial images. In this way, the authors create an object detector that reveals both objects of interest and adversarial patches. Although adversarial training is the most effective strategy to improve the stability of the model at the moment, it inevitably requires time-consuming training. It is expensive to use for large-scale datasets and complex deep architectures, and the emergence of new adversarial attacks leads to the retraining of neural network systems. To avoid this problem, many researchers attempt to remove the adversarial attack before passing it to the object detector.

There are a number of papers concerning the usage of adversarial training of external segmentator-based defenders. The PatchZero method [21] contains a segmentation model for detecting and removing adversarial patches. The Segment and Complete (SAC) method [22] consists of the well-proven U-Net architecture [23] for segmentation and removal of adversarial patches on input images. The authors of [24] proposed the Adversarial Pixel Masking (APM) method, in which the U-Net model is embedded in the general object detection pipeline. All these works contain an adversarially trained external segmentator. However, detectors in these methods also remain vulnerable to new attacks. The external defense models need to be trained on a huge variety of new adversarial examples, which again leads to the need to allocate large resources for training. The defense mechanism DIFfusion-based DeFender [25] is based on diffusion models and leverages the denoising capabilities of diffusion models to counteract adversarial patch attacks. The authors highlight the potential of generative models in enhancing adversarial robustness.

The self-adaptive learned Universal Defense Filter (UDFilter) [26] comprises two modules: an adversarial patch generation module and a defense filter generation module. This method weakens the negative impact of adversarial patches by applying the filter to the input image. In addition, the authors proposed a plug-and-play Joint Detection Strategy to maintain the model’s performance. The Patch-Agnostic Defense (PAD) method [27] is proposed to localize and remove adversarial patches without prior knowledge about the attack. The PAD includes the Segment Anything Model [28] for more accurate segmenting of the patches. The Local Gradient Smoothing (LGS) method [29] is independent of the patch attack, where the gradient of the input adversarial image is calculated to localize the adversarial patch. It is known that the gradient allows one to determine the image area containing high frequencies.

In recent years, some researchers have offered certified defensive methods against adversarial patches. For example, the Detectorguard method [30] detects attacks without removing them. It may lead to the loss of model functionality during the attack. The Objectseeker method [31] removes parts of the input image. After that, the Objectseeker suggests at least one split of the input image in which the adversarial patch does not affect the detection of objects. However, when processing attack scenarios with numerous patches, this method may fail.

The TOP-ALCM method [32] represents a significant advancement in the field of video analysis for violence detection, which can be considered as abnormal behavior. By incorporating temporal object-based features and adaptive local context modeling, the method is able to detect violent behavior more effectively in challenging environments.

An analysis of the related work highlights an unsolved problem. Existing defense techniques against adversarial patch attacks offer important contributions to the security of object detection systems, but they still exhibit significant gaps. One major issue is that many of these methods are highly tailored to specific attack types or models, limiting their generalization across different attack strategies or detector architectures. Another challenge is the computational complexity of many existing defense strategies, which can be prohibitive in real-time applications, particularly in resource-constrained environments. The gradient-based methods are a promising approach for mitigating localized attacks, but they may not be effective when attackers specifically design patches to bypass gradient-based defenses. Consequently, a significant gap remains in efficient defense that provides broad coverage across diverse attack scenarios. Developing universal defense mechanisms that combine patch detection and mitigation of their influence is a key area for future research. This limits the applicability of existing methods to multiple variations of attacks. The main contribution of this paper is to solve this problem.

## 3. Materials and Methods

**Figure 2:**
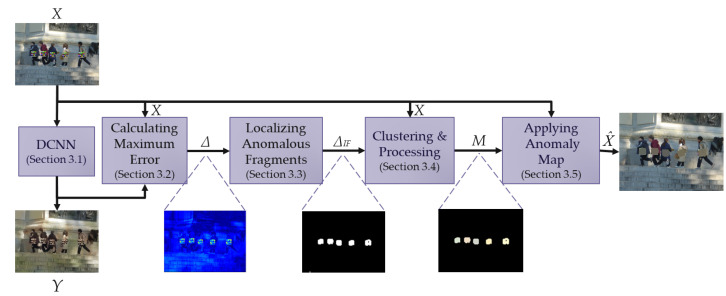
Simplified scheme of the proposed method.

The proposed method is based on detecting and removing anomalies in the input images. The differences between the benign image (image without adversarial attacks) and the adversarial image (image with adversarial attack) are considered anomalies in this work. A simplified scheme of the proposed method is shown in Figure 2.

The proposed algorithm is represented by a sequence of logical blocks implementing a specific task. An input image *X*, which can be either clear or adversarial, is fed to the input of the *Deep Convolutional Neural Network (DCNN)*. The *Deep Convolutional Neural Network (DCNN)* generates an approximate benign image *Y* based on the input image *X*. The *Calculating Maximum Error* block calculates the difference Δ between the input *X* and the generated *Y* images. The *Localizing Anomalous Fragments* block aims to extract anomalous fragments from the input images. The result of this block is the anomalous fragment map $\Delta_{IF}$, which is a binary matrix. The *Clustering and Processing* block groups the anomalies obtained in the previous step into clusters to process them further. The anomaly map *M* resulting from these blocks, which highlights areas with anomalies, is sent to the *Applying Anomaly Map* block to remove anomalous areas from the input image. The result of applying the anomaly map $\hat{X}$ is sent to the object detector. A more detailed description of each block from Figure 2 is shown below.

### 3.1. Deep Convolutional Neural Network for Benign Image Reconstruction

To solve the problem of the absence of a priori known benign images, we propose generating an approximately benign image based on the input one. Currently, the AnoGAN [33] addresses the problem of neural network-based image reconstruction. However, the AnoGAN architecture is quite simple and is capable of generating images that contain, for example, simple texture patterns and images of objects occupying a larger area in the image. It is worth noting that AnoGAN demonstrates high performance in localizing anomalous areas in medical images.

However, complex data are used in the present study, i.e., images with complex backgrounds and containing various multiple elements (people, road signs, vehicles). Also, the quality of these images may sometimes be insufficient. Thus, it is necessary to use a deep neural network architecture to reconstruct complex images, which leads to challenging training due to limited data.

To address this issue, we propose to use a Deep Convolutional Neural Network (DCNN in Figure 2), which, like in [33], is trained only on clean images in an unsupervised way. This approach allows obtaining a system that lacks knowledge of the attack, which increases its applicability to various attack variations.

The proposed DCNN architecture is inspired by the idea of an autoencoder [34]. So, the proposed DCNN consists of two parts: an encoder, which efficiently compresses the input data; and a decoder, which strives to reconstruct the original image from the compressed data. This aspect allows decreasing the content of the adversarial attack at the encoder stage and reconstructing the image from the compressed data with significant attack weakening. Additionally, to preliminarily decrease the attack, the DCNN is trained to convert a grayscale image to its colored version.

**Figure 3:**
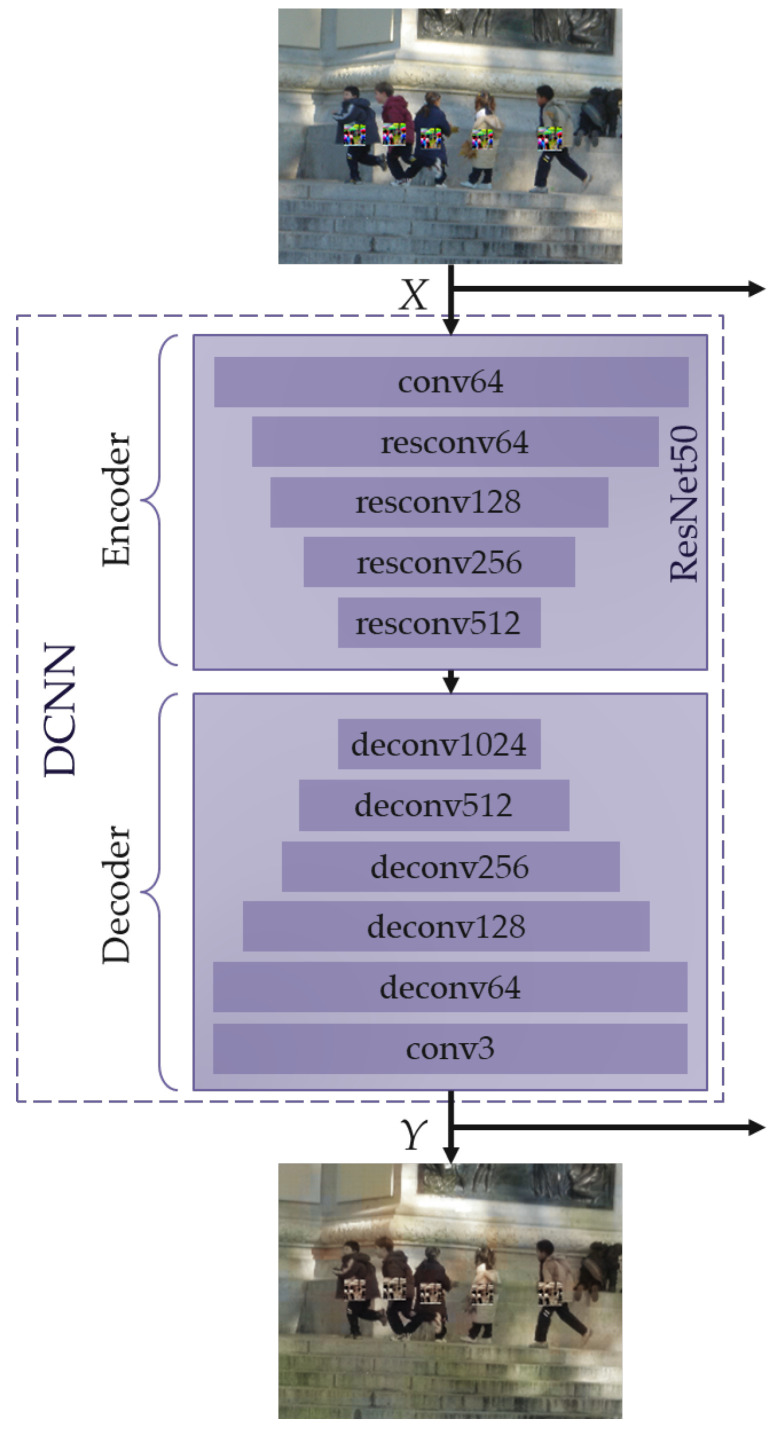
Simplified diagram of the proposed Deep Convolutional Neural Network (DCNN) architecture. The encoder is based on the convolutional part of ResNet50, excluding the final fully connected classification layer, and consists of sequential blocks that reduce the spatial dimensions of the input image. The decoder consists of sequential *deconvU* blocks, which increase the spatial dimensions to reconstruct the original image. The DCNN is trained using the mean squared error (MSE) loss function to minimize the difference between the original and reconstructed images. The input image *X* and the reconstructed image *Y* are processed through the blocks of the scheme presented in Figure 2.

Figure 3 shows a simplified diagram of the proposed DCNN. The fully convolutional part (without the last fully connected classification layer) of the deep neural network’s architecture, ResNet50 [35], is used as an encoder. This neural network consists of five blocks, each of which reduces the spatial dimensions of the input image by half. As in the paper [35], the conv64 block consists of a sequentially connected convolution layer with 64 output filters, a BatchNormalization layer, a ReLU activation layer, and a MaxPooling layer that reduces the spatial resolution by half. The *resconvU* block is a residual block with *U* output filters [35]. This part of the architecture directly follows the design of the original ResNet50 neural network, as described in the paper [35], with one modification: the final fully connected classification layer has been removed. This adjustment is made to ensure that the encoder outputs a compressed representation of the input image based on the input image.

As a decoder, we propose to use five sequentially connected blocks, each of which multiplies the spatial dimensions of the input data by two. The proposed *deconvU* block consists of sequentially connected transposed convolution layers with *U* output filters, a BatchNormalization layer, and a ReLU activation layer.

The mean squared error (MSE) function is used to calculate the difference between the original color image and the generated image [34]. MSE is then used as the loss function for training the proposed DCNN.

Thus, the proposed method for benign image reconstruction does not require labeling data, which makes this approach more flexible for solving multiple tasks. One can see that the proposed DCNN is quite simple, which makes it quite easy to reproduce this case. However, it is worth noting that the overall performance of the proposed method depends on the chosen DCNN architecture.

### 3.2. Calculating Maximum Error

**Figure 4:**
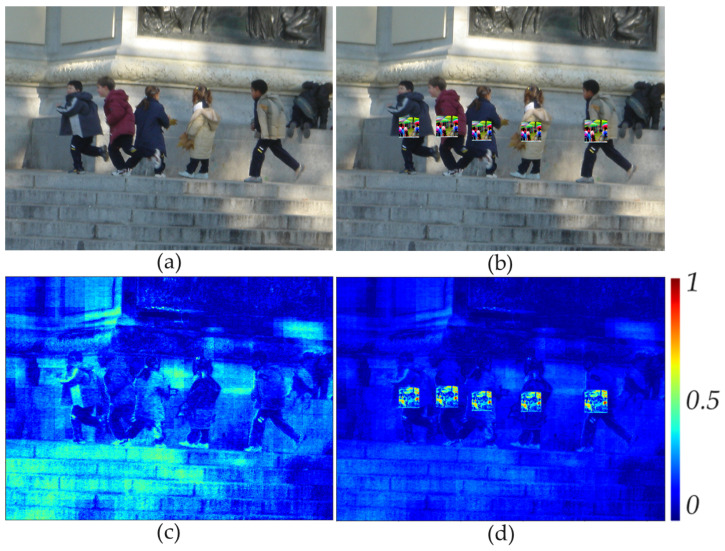
Example of a benign image (**a**) and its corresponding error map (**c**); example of an adversarial image (**b**) and its corresponding error map (**d**).

Let us denote the source color image as $X\in R^{H\times W\times 3}$, and the generated benign color image as $Y\in R^{H\times W\times 3}$. The spatial dimensions of the images are *H* and *W* pixels in height and width, respectively; the number of color channels is 3, which corresponds to the RGB format. The brightness value of the image pixels is normalized to the maximum possible pixel brightness, i.e., the values of *X* and *Y* vary in the range from 0.0 to 1.0. In this paper, the following expression is used to calculate the error value, representing the difference between the original and generated images:

(1) $$\Delta \left[i,j\right]=\max_{color}(|X\left[i,j,color\right]-Y\left[i,j,color\right]|),$$

where *i*, *j* are the indexes of the row and column of images, respectively; *color* is the index of the color channel of the image, which can take the values 0, 1, and 2 for the red, green. and blue channels, respectively; $\Delta \in R^{H\times W}$ is the error map between the source and generated benign images. Maximizing the error value for the color component allows reducing the number of parameters for subsequent processing, as well as to reduce information about the color reconstruction error. Figure 4 shows an example of an error map obtained by expression (1) for clean and adversarial images.

The areas with a patch attack that can be considered anomalies are visually highlighted in Figure 4d, i.e., the values of the error map are relatively high. It is also worth noting that Figure 4c shows errors in the reconstructed benign image, which should not be highlighted as anomalies. For these purposes, an anomalous fragments localizing algorithm was developed. This algorithm is described in the following section.

### 3.3. Localizing Anomalous Fragments

**Figure 5:**
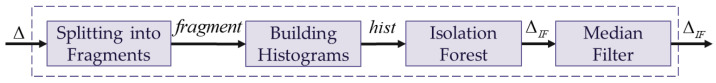
Simplified scheme of the *Localizing Anomalous Fragments* block.

A simplified scheme of the operation sequence in the *Localizing Anomalous Fragments* block is shown in Figure 5. This block consists of four consecutive sub-blocks, each of which operates a certain function. The error map Δ inputs to the *Split into Fragments* sub-block to obtain a new multi-dimensional matrix *fragment* by splitting the input two-dimensional error map Δ into fragments. The rich statistical data, such as histograms (named *hist* in Figure 5), are extracted from the input matrix by the *Building Histograms* sub-block. Further, the fragments’ histograms fed into the *Isolation Forest* sub-block obtain an anomalous fragments map $\Delta_{IF}$, which is a binary matrix. The ones in this binary matrix indicate the localization of anomalous fragments. The more detailed description of the *Localizing Anomalous Fragments* block is presented below.

In this paper, in order to identify anomalous areas, the error map is split into fragments by a sliding window of size F×F with a step S×S according to the following expression:

(2) $$\mathrm{fragment}[r,c,r_{F},c_{F}]=\Delta \left[r\cdot S+r_{F},c\cdot S+c_{F}\right],$$

where $\mathrm{fragment}\in R^{\frac{H}{S}\times \frac{W}{S}\times F\times F}$ is the result of splitting the error map into fragments; Δ is the error map from expression (1); *r* and *c* are the indexes of fragments in height and width, respectively; $r_{F}$ and $c_{F}$ are the indexes in height and width of each fragment, respectively.

Figure 4d shows that the error magnitude changes sharply at the patch attack location. The magnitude changes are smaller in the fragments without patch attack. Rich information about the change in errors within the fragments can be obtained from the histogram. Thus, for further processing of the error map, the values of the error map’s fragments transform into histograms. The described step can be formulated as the following expression:

(3) $$hist\left[r,c,b\right]=histogram(fragment\left[r,c,r_{F},c_{F}\right],B),$$

where $hist\in R^{\frac{H}{S}\times \frac{W}{S}\times B}$ is the result of calculating the histograms of all fragments; *B* is the number of bins to build a histogram; b∈{0,…,B−1} is the index of the bins; histogram(·) is the operator for calculating the histogram. The obtained histograms are fed into the Isolation Forest algorithm [36], which identifies fragments with anomalous histograms. Isolation Forest is an anomaly detection algorithm identifying anomalies by isolating data points. Anomaly points (outliers) are usually rare and have unique features and are easier to isolate from normal points. The algorithm uses a set of decision trees, which are structured to partition the data by splitting features. These trees sporadically partition the data points in random features, thus creating a tree structure. The required number of splits for data point isolation is calculated. Considering anomalous points are usually isolated in fewer splits than normal points, the Isolation Forest algorithm is efficient in detecting outliers. The Isolation Forest algorithm has linear time complexity to the number of samples and features, and is scalable to large datasets. The algorithm is robust to noise and is applicable for non-linearly separable data distributions. As normal fragments (with no perturbations or adversarial patches) have predictable histogram form, the Isolation Forest algorithm is also efficient in detecting histogram outliers. So, the Isolation Forest algorithm is a powerful tool for identifying anomalous data. Thus, a sharp change in the histograms of fragments where the patch attack is located, relative to the other histograms, can be considered anomalous behavior. This anomalous behavior is detected by the Isolation Forest algorithm. This stage can be represented by the following formula:

(4) $$\Delta_{IF}=IsolationForest\left(hist\right),$$

where $\Delta_{IF}\in {\left\{0,1\right\}}^{\frac{H}{S}\times \frac{W}{S}}$ is the obtained anomalous fragments map, which is a binary matrix with spatial dimensions $\frac{H}{S}\times \frac{W}{S}$ such that

(5) $$\Delta_{IF}\left[r,c\right]=\left\{\begin{array}{cc} 1 & \mathrm{if}\;\mathrm{fragment}\left[r,c\right]\;\mathrm{is}\;\mathrm{anomalous},\\ 0 & \mathrm{elsewise}. \end{array}\right.$$

**Figure 6:**
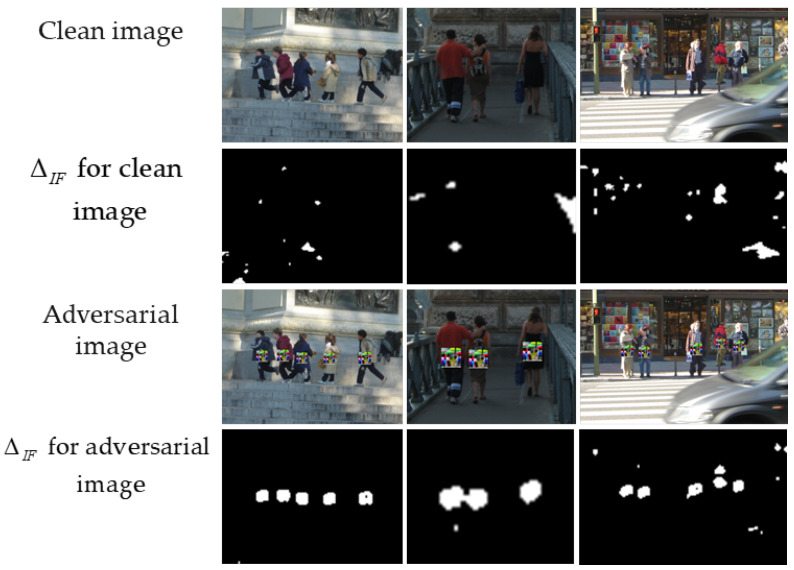
Examples of anomalous fragment maps $\Delta_{IF}$ for clean images (the second row) and for adversarial images (the fourth row).

A median filter is applied to remove scattered fragments marked as anomalous while simultaneously grouping dense anomalous fragments. Figure 6 shows results of the anomalous fragment localization algorithm for the clean and adversarial images mentioned.

The algorithm described above highlights anomalous fragments, as one can see from Figure 6. However, due to the lack of a priori knowledge about attacks (their presence, number, size, and shape), it is necessary to post-process the obtained information. The post-processing algorithm is described in the next subsection.

### 3.4. Clustering and Processing of the Anomalies

**Figure 7:**
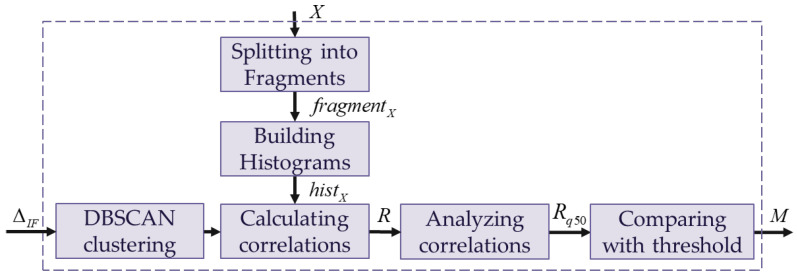
Simplified scheme of the proposed clustering and processing block.

A simplified scheme of the proposed *Clustering and Processing* block is presented in Figure 7. This block includes two paths. The vertical path processes the input image *X* (see Figure 2) and consists of two sub-blocks: *Splitting into Fragments* and *Building Histograms*. *Splitting into Fragments* splits the input image *X* into fragments to obtain the multi-dimensional matrix $fragment_{X}$. *Building Histograms* extracts histograms $hist_{X}$ for each fragment from matrix $fragment_{X}$. The horizontal path includes four sub-blocks to process the anomalous fragments map $\Delta_{IF}$. The *DBSCAN clustering* sub-block groups anomalous fragments into clusters. These groups and the multi-dimensional matrix $hist_{X}$ are fed to *Calculating correlations* sub-block simultaneously to obtain the correlation coefficients between the fragments’ histograms $hist_{X}$ that indicates anomalous areas and the nearest normal areas. Further, the calculated correlation coefficients pass through the *Analyzing correlations* and *Comparing with threshold* sub-blocks to obtain an anomaly map *M* that is more accurate than the input anomalous fragments map $\Delta_{IF}$. The more detailed description of the *Clustering and Processing* block is presented below.

The coordinates of anomalous fragments obtained in the previous stage are fed as input data into the DBSCAN clustering algorithm [37]. The DBSCAN algorithm solves the problem of grouping closely located anomalous fragments and removing randomly marked anomalous fragments. The DBSCAN clustering algorithm allows for finding groups of densely located points without prior knowledge of the number and shapes of these groups, and, moreover, the DBSCAN algorithm is robust to noise outliers, which may occur in the case of insufficiently accurate image generation in the DCNN, for example. As a result of the clustering stage, *L* clusters are obtained, and each cluster contains a different number of fragments belonging to a specific cluster.

The next stage is calculating the Pearson correlation coefficient [38] between the histograms of fragments belonging to the cluster with index *l* and the histograms of neighbor fragments around the cluster with index *l*. The correlation coefficient is a scalar that indicates the level of relationship between two variables. The correlation coefficient is convenient due to its clear interpretation: it approaches 1.0 if the compared data are very similar; it approaches 0.0 if the compared data are completely different.

At this stage, the histograms of the fragments from the source image *X* are analyzed. Initially, the source image *X* is split into fragments according to the same rule as in the block for localizing anomalous fragments:

(6) $$fragment_{X}\left[r,c,r_{F},c_{F},color\right]=X\left[r\cdot S+r_{F},c\cdot S+c_{F},color\right],$$

where $fragment_{X}\in R^{\frac{H}{S}\times \frac{W}{S}\times F\times F\times 3}$ is the result of splitting the source image *X* into fragments by sliding window with size F×F and stride S×S, *and color* is the index of the color channel. Further, as in the localizing anomalous fragments block, a histogram is built for each fragment according to the following rule:

(7) $$hist_{X}\left[r,c,b_{X}\right]=histogram\left(fragment_{X}\left[r,c,r_{F},c_{F},color\right],B_{X}\right),$$

where $hist_{X}\in R^{\frac{H}{S}\times \frac{W}{S}\times B_{X}}$ is the result of calculating the histograms of all fragments in $fragment_{X}$, $B_{X}$ is the number of bins to build a histogram, and $b_{X}\in \left\{0,\ldots ,B_{X}-1\right\}$ is the index of the bins.

The result of the *Calculating correlations* algorithm for each cluster with index *l* is a two-dimensional matrix, each element of which is calculated as follows:

(8) $$R\left[l,a_{l},n_{l}\right]=PearsonCorrelationCoefficient\left(hist_{Xanomalous}\left[a_{l}\right],hist_{Xnormal}\left[n_{l}\right]\right),$$

where $hist_{Xanomalous}\in R^{A\times B_{X}}$ and $hist_{Xnormal}\in R^{N\times B_{X}}$ are the sets of the histograms, built from the source image’s fragments, identified as anomalous and normal at the localizing anomalous fragments block, respectively; *A* is the number of anomalous fragments; *N* is the number of normal fragments; $a_{l}$ is the index of the anomalous fragment in the cluster with index *l*; and $n_{l}$ is the index of the normal fragment, which is in the neighborhood around the cluster with index *l*. As a result of executing this algorithm, a set of two-dimensional Pearson correlation coefficient matrices for each cluster is obtained.

Given that the adversarial patch attack represents a local perturbation, it can be logically concluded that the image around the adversarial patch attack is semantically different from the image of the patch attack itself (these two images do not correlate).

It can be concluded that the histograms of adversarial patch attacks should differ significantly from the histograms of the neighborhood around this patch attack. Since the correlation coefficient indicates the level of similarity, the correlation coefficient between the adversarial patch attack and the neighborhood around this attack should be low due to the semantic independence of the patch attack and its neighborhood. Thus, for each cluster, the median value of the Pearson correlation coefficient is calculated according to the following rules:

(9) $$R_{q50}\left[l,a_{l}\right]=\underset{n_{l}}{median}\left(R\left[l,a_{l},n_{l}\right]\right),$$

(10) $$R_{q50}\left[l\right]=\underset{a_{l}}{median}\left(R_{q50}\left[l,a_{l}\right]\right),$$

where $\underset{n_{l}}{median}\left(\cdot \right)$ and $\underset{a_{l}}{median}\left(\cdot \right)$ are the operations that calculate the median value along a certain variable. The obtained median value of the Pearson correlation coefficient is compared with the empirically established threshold. If this value is lower than the threshold, the cluster is considered anomalous.

In this paper, to obtain an anomaly map *M* (see Figure 2), the pixel brightness values from an anomalous cluster are extracted from the source image *X* and their average value is calculated. The average pixel value in the anomalous cluster is assigned to pixels in the anomaly map *M* at the location of this cluster.

**Figure 8:**
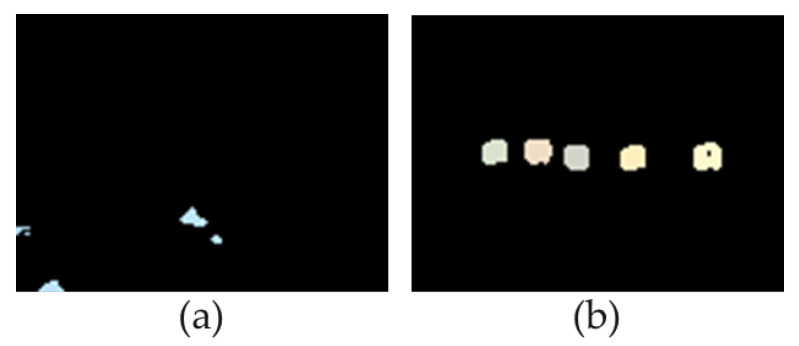
Examples of anomaly maps for a benign image (**a**) and for an adversarial image (**b**).

Figure 8 shows examples of anomaly maps for the clean and adversarial images mentioned in Figure 4a,b, respectively. The maps are obtained as a result of the clustering and processing algorithm.

Figure 8 shows that the algorithm described above processes the data extracted from the localizing anomalous fragments block and makes a decision to identify these data as anomalous or not.

### 3.5. Applying an Anomaly Map to an Image

The obtained anomaly map must be applied to the source image *X* as follows:

(11) $$\hat{X}\left[i,j\right]=\left\{\begin{array}{cc} M[i,j] & \mathrm{if}\;M[i,\;j]>0,\\ X[i,j] & \mathrm{elsewise}. \end{array}\right.,$$

The expression (11) allows to save the source image hiding anomalous areas. The obtained image $\hat{X}$ is sent to the attacked object detection system as input for subsequent objects of interest detection. Thus, the localized adversarial patch attacks in the image are replaced by uniformly colored figures, which reduce the strength of the attacks to a minimum, thereby minimizing their impact on the object detection system.

## 4. Results

### 4.1. Implementation Details

The YOLOv3 [8] pre-trained using MS COCO 2017 is used as an object detector. The MS COCO dataset [9] is a widely used benchmark dataset in computer vision, specifically designed for object detection, segmentation, and captioning tasks. It includes over 80 object categories (including “people” category) and contains over 330,000 images, with around 200,000 labeled and over 1.5 million object instances. The MS COCO dataset is publicly available for research purposes. The DCNN proposed in Section 3.1 was trained using only the part of the MS COCO training dataset that has images containing people. Let us name this dataset as *MS COCO Person*. The volume of the MS COCO Person training dataset is 64,115 images. The DCNN was trained for 48 epochs; the batch size is 4. The learning rate first warms up to a value of 1 $\times \;10^{-4}$ in 500 iterations, after which it decreases to a value of 1 $\times \;10^{-5}$ according to the cosine schedule. The optimizer is Adam with $\beta_{1}=0.9$ and $\beta_{2}=0.99$.

In Section 3.3, a sliding window has a size F = 8 and a stride S = 8.

The Isolation Forest and DBSCAN algorithms used in Section 3.4 are implemented in the Python v.3.8.10 programming language using the scikit-learn package [39]. The DBSCAN algorithm requires setting two parameters: the minimum number of points minPts is 9 and the size of the neighborhood ϵ is $2\sqrt{2}$ to obtain dense clusters. The remaining parameters for the Isolation Forest and DBSCAN algorithms are taken by default from the scikit-learn package. The threshold of the median Pearson coefficient in Section 3.4 is 0.1.

The experiments were conducted using a single NVIDIA (Santa Clara, CA, USA) Tesla V100 GPU, Python programming language, TensorFlow framework, and Scikit-learn package on an Ubuntu 20.04 operating system.

### 4.2. Experimental Results

The most popular methods aimed at defending pedestrian detectors from adversarial patch attacks, namely, the PAD [27] and the SAC [22], are used for comparison. It is also worth noting that the implementations of these methods are publicly available, which makes it possible to compare them using the same test dataset and object detector.

Initially, the SAC method was applied to detect the digital patch attack projected gradient descent (PGD) [40], using self-training for this purpose. However, the analysis of the effectiveness of the SAC method on patch attack images from work [13] did not yield positive results. For this experiment, the SAC method was trained on the MS COCO Person training dataset with applied real-world adversarial patch attacks from [13]. The other settings for this method remained unchanged.

Table 1 presents a comparative analysis of the proposed and the existing patch attack defense methods. The object detection quality metrics mAP [9] and ASR were calculated on the INRIA-Person [14] test dataset without adding attacks (*Clean* column) and with adding attacks (*Adversarial* column). The Attack Success Rate (ASR) quantifies the proportion of adversarial examples that successfully deceive a machine learning model into making an incorrect prediction or classification. It is a key metric in adversarial robustness research, where the goal is to understand how well a model can resist adversarial perturbations. A high attack success rate indicates that the model is highly vulnerable to adversarial perturbations and can be easily fooled. A low attack success rate suggests that the model is more robust and resistant to adversarial attacks. [41]. The ASR can be expressed as follows:

(12) $$ASR=\frac{O_{clean}-O_{adv}}{O_{clean}},$$

where $O_{adv}$ denotes the sum of detected objects in the adversarial image; $O_{clean}$ denotes the sum of detected objects in the clean image in the test dataset.

The INRIA-Person dataset [14] is a benchmark for pedestrian detection in computer vision. It provides a dataset with samples of annotated pedestrians, primarily in urban environments. Its simplicity and relevance have made it a widely used resource for training and evaluating detection algorithms. The test dataset consists of 288 images and 597 pedestrian samples.

The results presented in Table 1 show that the proposed method outperforms the state-of-the-art SAC and PAD methods.

**Figure 9:**
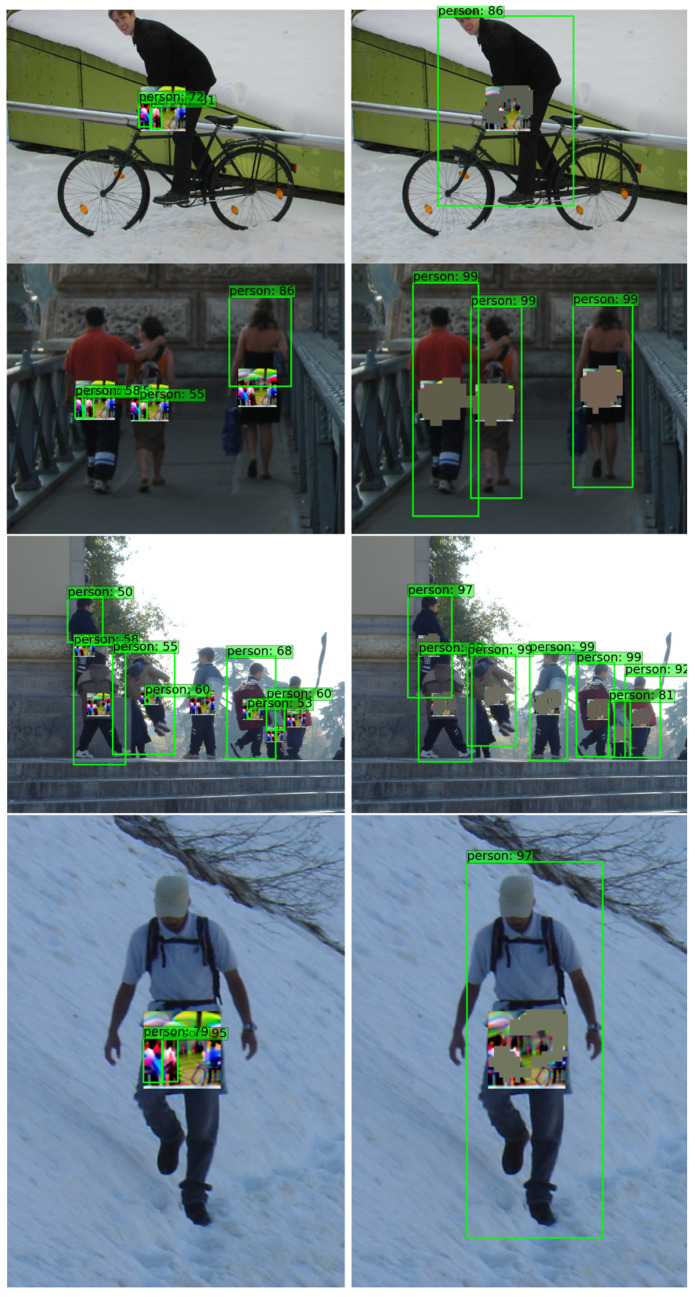
Visualization of YOLOv3 object detection on several examples from the INRIA-Person dataset, subjected to an adversarial patch attack [13]. The green bounding boxes represent detected “person” objects, with the objectness score displayed in the top-left corner of each box. The left column shows detection results without defensive pre-processing, while the right column illustrates the impact of the proposed defense method.

Figure 9 illustrates the YOLOv3 model described in Section 4.1. It presents several examples from the INRIA-Person dataset, to which an adversarial patch attack [13] has been applied. The green bounding boxes indicate the detected objects classified as “person”, with the top-left corner of each box displaying the object category and its corresponding objectness score. The left column of the figure shows the object detection results in images without any defensive pre-processing, while the right column displays the results after applying the proposed defense method.

The results presented in Figure 9 clearly demonstrate the impact of the adversarial patch attack on the YOLOv3 object detection system. It is evident that the adversarial patch degrades the performance of the detector by reducing the objectness scores of the detected “person” objects, effectively hiding them. Additionally, the attack deceives the neural network, leading to altered predictions, as shown in the left column of Figure 9. In contrast, the right column in Figure 9 illustrates the effectiveness of the proposed defense method, which successfully localizes the patch attack and mitigates its negative effects on the predictions of the YOLOv3. This highlights the ability of the defense to preserve detection accuracy in the presence of adversarial perturbations.

The existing SAC [22] method required prior knowledge of the attack in order to train the network to segment these attacks. This aspect leads to decreasing the ability to recognize a novel type of patch attack. The PAD [27] method does not require knowledge of the attack. The PAD method uses semantic independence and spatial heterogeneity obtained from the image. The proposed method includes the training-base part to process complex data exactly, images with an abundant background and foreground with a lot of objects on it, and statistical analysis to decrease the randomness factor. So, the proposed method is considered a powerful tool in computer vision tasks. Also, the proposed method does not require knowledge of a new attack, which does not lead to the necessity for retraining the neural network component.

## 5. Discussion

A new method for increasing the robustness of the neural-based pedestrian detector is presented in this paper. The method aims to counteract adversarial patch attacks by localizing anomalies in the images. The proposed method consists of a deep convolutional neural network, an anomaly localizing algorithm, and a clustering and anomaly processing algorithm.

The DCNN generates the image that is close to the input image but with reduced presence of adversarial attacks. This aspect excludes the necessity to retrain the object detector when new examples of adversarial patch attacks appear.

The proposed method utilizes rich statistical information obtained from the local histograms of the error map and input images. The combination of the aforementioned conventional data processing algorithms represents a powerful tool for counteracting the local anomalous perturbations in images.

This paper also demonstrates that even a relatively simple DCNN architecture, combined with the proposed anomaly localizing and post-processing algorithm, outperforms the state-of-the-art methods, namely, SAC and PAD. The idea of using a lightweight deep convolutional neural network and conventional machine learning methods allows for the effective processing of complex images without high computational costs.

The proposed method also does not use any knowledge about patch attacks and does not require retraining the object detector, and is thus computationally efficient. It makes the proposed method suitable for a variety of applications, which utilize relatively simple and cheap hardware.

## 6. Conclusions and Future Work

In this paper, we introduced a novel method to enhance the robustness of neural-based pedestrian detectors against adversarial patch attacks by using anomaly localization techniques. Our approach effectively combines deep convolutional neural networks, statistical anomaly detection, and clustering to identify and mitigate adversarial perturbations. Experimental evaluations on the INRIA-Person dataset demonstrated the method’s effectiveness, with significant improvements in detection performance under adversarial conditions, achieving a notable increase in mAP50 score compared to an unprotected baseline. The proposed method is highly efficient and adaptive; therefore, it can be used in various image localization and detection systems implemented on a variety of hardware platforms.

Despite its promising results, the proposed method has some limitations that require further study. One key drawback is the increased computational complexity introduced by components such as histogram-based anomaly detection and clustering. These processes can slightly decrease the method’s efficiency in real-time high resolution video processing. Future work could focus on optimizing these processes through model compression, efficient algorithm design, or hardware acceleration to enable real-time performance without losing detection accuracy.

The performance of the method relies on parameters and thresholds set during anomaly detection and clustering, which may not generalize well across various datasets or detection tasks. Adaptive mechanisms that can automatically adjust these parameters based on input characteristics would enhance the method’s robustness and versatility.

The proposed approach has been tested only in the domain of pedestrian detection using YOLOv3. Its effectiveness in other application domains is still unverified. Furthermore, we should note that the proposed technique may be ineffective against natural perturbations, such as severe brightness/contrast changes, image blur, large blind spots (sun/laser radiation), and highly compressed images (domestic shifts). In future work, we will be extending our research to various CNN-based localization and detection applications (aerial imaging, medical imaging, road signs detection, etc), providing results for a broader set of state-of-the-art CNNs/clustering/isolation algorithms.

By addressing these limitations, the proposed method could evolve into a more efficient, adaptable, and universally applicable defense against adversarial attacks, significantly strengthening the security of neural-based detection systems.

In conclusion, the proposed anomaly localization-based method represents a significant step toward improving the security of object detection systems against adversarial patch attacks. By addressing its computational challenges, exploring robustness under variety of attack types, and extending its applicability to new domains, this work can contribute to the development of more resilient and versatile object detection pipelines.

## Figures and Tables

**Table 1:** mAP(%) and ASR(%) under adversarial patch attack [13] for different defense methods. The best performance is bolded, and the suboptimal performance is underlined.

Defense Methods	Clean	Adversarial	ASR
Undefended	93.35	46.79	19.42
SAC [22]	93.35	53.72	10.61
PAD [27]	92.73	76.94	11.76
**Proposed**	**93.55**	**80.97**	**8.40**

## Data Availability

Publicly available datasets were used in this study.
